# Ghost Authorship in Industry-Initiated Randomised Trials

**DOI:** 10.1371/journal.pmed.0040019

**Published:** 2007-01-16

**Authors:** Peter C Gøtzsche, Asbjørn Hróbjartsson, Helle Krogh Johansen, Mette T Haahr, Douglas G Altman, An-Wen Chan

**Affiliations:** 1 Nordic Cochrane Centre, Copenhagen, Denmark; 2 Centre for Statistics in Medicine, Oxford, United Kingdom; 3 Department of Medicine, University of Toronto, Canada; United Kingdom

## Abstract

**Background:**

Ghost authorship, the failure to name, as an author, an individual who has made substantial contributions to an article, may result in lack of accountability. The prevalence and nature of ghost authorship in industry-initiated randomised trials is not known.

**Methods and Findings:**

We conducted a cohort study comparing protocols and corresponding publications for industry-initiated trials approved by the Scientific-Ethical Committees for Copenhagen and Frederiksberg in 1994–1995. We defined ghost authorship as present if individuals who wrote the trial protocol, performed the statistical analyses, or wrote the manuscript, were not listed as authors of the publication, or as members of a study group or writing committee, or in an acknowledgment. We identified 44 industry-initiated trials. We did not find any trial protocol or publication that stated explicitly that the clinical study report or the manuscript was to be written or was written by the clinical investigators, and none of the protocols stated that clinical investigators were to be involved with data analysis. We found evidence of ghost authorship for 33 trials (75%; 95% confidence interval 60%–87%). The prevalence of ghost authorship was increased to 91% (40 of 44 articles; 95% confidence interval 78%–98%) when we included cases where a person qualifying for authorship was acknowledged rather than appearing as an author. In 31 trials, the ghost authors we identified were statisticians. It is likely that we have overlooked some ghost authors, as we had very limited information to identify the possible omission of other individuals who would have qualified as authors.

**Conclusions:**

Ghost authorship in industry-initiated trials is very common. Its prevalence could be considerably reduced, and transparency improved, if existing guidelines were followed, and if protocols were publicly available.

## Abstract 2

Of 44 industry-initiated trials, there was evidence of ghost authorship in 33, increasing to 40 when a person qualifying for authorship was acknowledged rather than appearing as an author.

## Editors' Summary

**Background.:**

Original scientific findings are usually published in the form of a “paper”, whether it is actually distributed on paper, or circulated via the internet, as this one is. Papers are normally prepared by a group of researchers who did the research and are then listed at the top of the article. These authors therefore take responsibility for the integrity of the results and interpretation of them. However, many people are worried that sometimes the author list on the paper does not tell the true story of who was involved. In particular, for clinical research, case histories and previous research has suggested that “ghost authorship” is commonplace. Ghost authors are people who were involved in some way in the research study, or writing the paper, but who have been left off the final author list. This might happen because the study “looks” more credible if the true authors (for example, company employees or freelance medical writers) are not revealed. This practice might hide competing interests that readers should be aware of, and has therefore been condemned by academics, groups of editors, and some pharmaceutical companies.

**Why Was This Study Done?:**

This group of researchers wanted to get an idea of how often ghost authorship happened in medical research done by companies. Previous studies looking into this used surveys, whereby the researchers would write to one author on each of a group of papers to ask whether anyone else had been involved in the work but who was not listed on the paper. These sorts of studies typically underestimate the rate of ghost authorship, because the main author might not want to admit what had been going on. However, the researchers here managed to get access to trial protocols (documents setting out the plans for future research studies), which gave them a way to investigate ghost authorship.

**What Did the Researchers Do and Find?:**

In order to investigate the frequency and type of ghost authorship, these researchers identified every trial which was approved between 1994 and 1995 by the ethics committees of Copenhagen and Frederiksberg in Denmark. Then they winnowed this group down to include only the trials that were sponsored by industry (pharmaceutical companies and others), and only those trials that were finished and published. The protocols for each trial were obtained from the ethics committees and the researchers then matched up each protocol with its corresponding paper. Then, they compared names which appeared in the protocol against names appearing on the eventual paper, either on the author list or acknowledged elsewhere in the paper as being involved. The researchers ended up studying 44 trials. For 31 of these (75% of them) they found some evidence of ghost authorship, in that people were identified as having written the protocol or who had been involved in doing statistical analyses or writing the manuscript, but did not end up listed in the manuscript. If the definition of authorship was made narrower, and “ghost authorship” included people qualifying for authorship who were mentioned in the acknowledgements but not the author list, the researchers' estimate went up to 91%, that is 40 of the 44 trials. For most of the trials with missing authors, the ghost was a statistician (the person who analyzes the trial data).

**What Do These Findings Mean?:**

In this study, the researchers found that ghost authorship was very common in papers published in medical journals (this study covered a broad range of peer-reviewed journals in many medical disciplines). The method used in this paper seems more reliable than using surveys to work out how often ghost authorship happens. The researchers aimed to define authorship using the policies set out by a group called the International Committee of Medical Journal Editors (ICMJE), and the findings here suggest that the ICMJE's standards for authorship are very often ignored. This means that people who read the published paper cannot always accurately judge or trust the information presented within it, and competing interests may be hidden. The researchers here suggest that protocols should be made publicly available so that everyone can see what trials are planned and who is involved in conducting them. The findings also suggest that journals should not only list the authors of each paper but describe what each author has done, so that the published information accurately reflects what has been carried out.

**Additional Information.:**

Please access these Web sites via the online version of this summary at http://dx.doi.org/10.1371/journal.pmed.0040019.

- Read the Perspective by Liz Wager, which discusses these findings in more depth
- The International Committee of Medical Journal Editors (ICMJE) is a group of general medical journal editors who have produced general guidelines for biomedical manuscripts; their definition of authorship is also described
- The Committee on Publication Ethics is a forum for editors of peer-reviewed journals to discuss issues related to the integrity of the scientific record; the Web site lists anonymized problems and the committee's advice, not just regarding authorship, but other types of problems as well
- Good Publication Practice for Pharmaceutical Companies outlines common standards for publication of industry-sponsored medical research, and some pharmaceutical companies have agreed to these

## Introduction

Authorship establishes accountability, responsibility, and credit for scientific articles [1]. If authorship is misappropriated, readers may be misled, and the potential for manipulated analyses and conclusions may increase. One type of misappropriation is ghost authorship, which has been defined as the failure to name, as an author, an individual who has made substantial contributions to the research or writing of the article [1].

A confidential survey of corresponding authors of research reports, editorials, reviews, and opinion articles in six medical journals with 69% response rate showed that 13% of 809 articles had ghost authors [1]. This is likely an underestimate because of the modest response rate and because those who responded might be reluctant to admit that ghost authors had contributed to their paper.

We examined directly the prevalence and nature of ghost authorship in a cohort of industry-initiated randomised trials by comparing the trial protocols with subsequent publications. We have previously documented widespread constraints on the publication rights of clinical investigators in these trials [2].

## Methods

For all published industry-initiated randomised trials approved in 1994–1995 by the Scientific-Ethical Committees for Copenhagen and Frederiksberg in Denmark, we compared the full trial protocols with the publications. We initially identified 274 approved trial protocols, but after extensive literature searches in MEDLINE, EMBASE, and the Cochrane Controlled Trials Register, and a survey of the trialists in 2003, we found that 172 trials (63%) were never begun, completed, or published [3]. Of the 102 published trials, 56 had industry support [3], but in some cases this was rather minor, such as delivering coded drugs to a trial that was initiated by academic researchers. Since we wished to study industry-initiated trials, we excluded 12 such trials that were initiated by the investigators.

When there was more than one publication for a trial, we used the one that reported the results for the primary outcomes, and if no primary outcome was defined, the first publication that reported final results. The median publication year was 1999 (range from 1997 to 2002).

Two observers independently extracted data from each protocol or publication on name and nationality of the sponsor; type and location of the trial; and the roles of the investigators and sponsor in trial design, data collection, data analysis and interpretation, and manuscript preparation, as noted anywhere in the text, including separate agreements in trial protocols as well as footnotes and acknowledgments in publications using an electronic form. For some items, the observer could add extracted quotations or write comments. To reduce redundancy, the second observer was provided with this additional text. The same observer did not review both the protocol and the report for the same trial; there was no other blinding. Disagreements were resolved by discussion.

We defined ghost authorship as present if individuals who wrote the trial protocol, performed the statistical analyses, or wrote the manuscript, were not listed as authors of the publication, or as members of a study group or writing committee, or in an acknowledgment.

These criteria were an operationalisation of the guidelines published by the International Committee of Medical Journal Editors [4], which state that “an author is someone who has made substantive intellectual contributions to a published study.” Furthermore, authorship credit should be based on “(1) substantial contributions to conception and design, or acquisition of data, or analysis and interpretation of data; (2) drafting the article or revising it critically for important intellectual content; and (3) final approval of the version to be published.” Authors should meet all three conditions; all persons designated as authors should qualify for authorship; and all those who qualify should be listed. These guidelines are also recommended by The Pharmaceutical Research and Manufacturers of America [5].

We believe that all who contribute in a major way under both (1) and (2) should be authors. As it is also likely that some of those who have only been acknowledged might have contributed sufficiently under either (1) or (2) to have merited authorship, we did an additional analysis where people who were acknowledged for having performed the statistical analyses or having written the protocol or the manuscript were considered ghost authors.

## Results

A total of 44 trials were included, of which 43 (98%) were initiated by one of 26 multinational pharmaceutical firms and one by a local company. Of the total trials, 33 were multicentre and multinational, two were multicentre Danish trials, and nine were single-centre trials.

We found evidence of ghost authorship for 31 of the 44 trials (75%; 95% confidence interval 60%–87%) (Table 1). If individuals who qualified for authorship, but who were acknowledged rather than listed as authors, were considered ghost authors, the prevalence of ghost authorship was 91% (95% confidence interval 78%–98%). In 31 trials, the ghost authors we identified were statisticians. A total of eight publications acknowledged the assistance of statisticians, and four acknowledged the assistance of medical writers (Table 1).

**Table 1:**
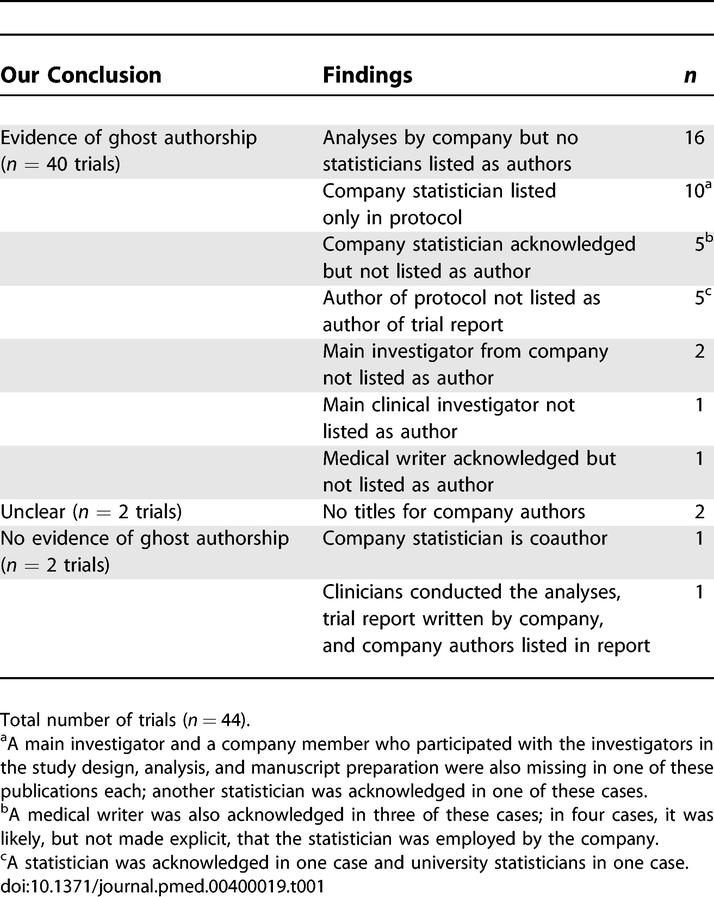
Ghost Authorship in Trial Reports

It was explicitly stated in 26 protocols that the company would conduct the statistical analyses or write the clinical study report or the manuscript. We did not find any trial protocol or publication stating explicitly that the study report or the manuscript was to be written or was written by the clinical investigators, and none of the protocols stated that clinical investigators were to be involved with data analysis. In three cases, clinicians participated in end-point or clean file committees, and in three other cases, clinicians decided together with the sponsor which data should be excluded from analysis.

It was also unclear whether clinicians had contributed to the protocols. None of the trial protocols described explicitly who had contributed to the design of the trial. In two protocols a group of clinicians was mentioned who were to advise the company on protocol design. Only five protocols explicitly identified the author of the protocol, but none of these individuals—all of whom were company employees—were listed as authors of the publications or were thanked in the acknowledgments, although one protocol had noted that the “author of this protocol will be included in the list of authors.”

A total of 15 reports had named authors and a study group, and three reports were authored by a study group and identified a writing committee. There were three other reports that had no authors and no writing committee, and it was therefore not possible to know who of the many clinicians listed as members of the study group had been authors. The remaining 21 reports listed the authors in the byline. Only one publication had a description of contributorship, and only one a conflict of interest statement.

All published reports had clinicians as authors. Company employees were listed among the authors for 28 (64%) of the 44 publications; there were no such authors for 12 publications. It was not clear whether some of the authors for the remaining four reports were company employees as names were listed without affiliations.

## Discussion

We found a high prevalence of ghost authorship in industry-initiated randomised trials. To our knowledge, this study is the first that has systematically examined the prevalence of ghost authorship using a cohort of protocols and corresponding publications. We defined ghost authorship as present if an individual who wrote the trial protocol, or performed the statistical analyses, or wrote the manuscript did not appear among the authors, or members of a study group or writing committee, or in an acknowledgment. Our criteria are similar to those used in two previous surveys of authors [1,6], and by these criteria we found evidence of ghost authorship in 75% of the articles. In contrast, these two surveys, which relied on self-reporting, found rates of ghost authorship of 13% [1] and 11% [6], respectively. The latter survey addressed 362 Cochrane Reviews; such reviews are a special case since there is often far more collaboration between editors and authors than for other articles. Most commonly, it was a member of the Cochrane editorial team who was judged to have deserved authorship [6], but it should be noted that journal editors are generally discouraged from becoming authors of the manuscripts they edit because of the obvious conflict of interest. We are not aware of other studies of the prevalence of ghost authorship.

It is a strength of our study that our sample is representative of all industry-initiated clinical trials, as it covers a wide range of diseases and specialties, and involves large multinational companies. Furthermore, the trials were generally published in well-known peer-reviewed journals (Table 2), and not in supplements to such journals.

**Table 2:**
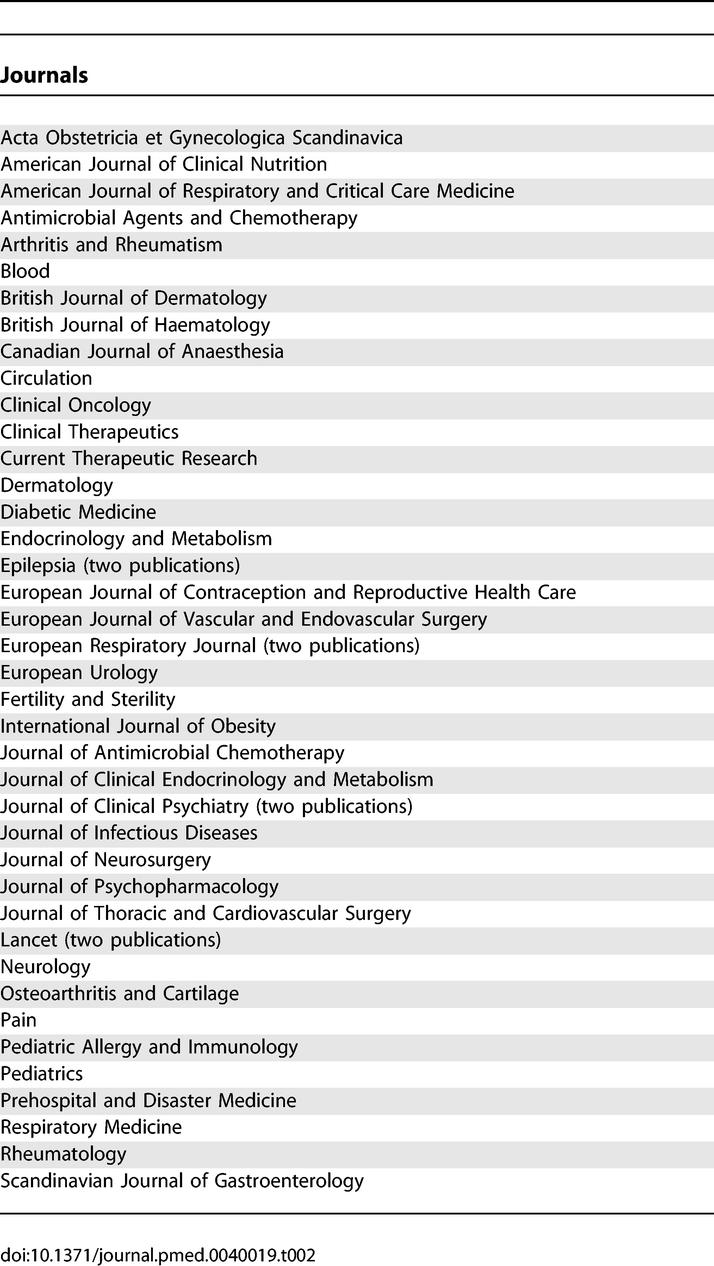
List of Journals in which the Results for the Primary Outcomes from the 44 Trials Were Published

The small sample size is a limitation of our study. Another limitation is the dates for the protocols, 1994–1995. However, as the median publication year for all the studies was 1999, it is not likely that the situation would be much different today. A number of well-intentioned guidelines have appeared recently, but we found that constraints on the publication rights of clinical investigators in protocols for industry-initiated trials from 2004 were similar to those in protocols from 1994 to 1995 [2].

It can be questioned whether the main investigator from the company (two cases) or the main clinical investigator (one case) should necessarily become authors (Table 1). On the other hand, it is likely that we have not identified all ghost authors, as we had very limited information with which to identify the possible omission of other individuals who would have qualified as authors, other than statisticians. We found only three publications (7%) that had statisticians among the authors, in two cases from the company and in the third from a university, although it was explicitly stated in 26 protocols that the company conducted the statistical analyses or wrote the clinical study report or the manuscript. This might have been the case for all trials, since the companies collected the data and are obliged by law to submit a report of the results to the Danish Drug Agency. These findings are in sharp contrast to a survey, with a 75% response rate, of 704 authors of manuscripts submitted to the *BMJ* and the *Annals of Internal Medicine* (only a minority of which were randomised trials), which found that statisticians and similar methodologists who had made a significant contribution at some stage of the research process were authors in 86% of the cases [7].

We take issue with this widespread practice of not including statisticians as authors for reports of randomised trials. Multicentre trials are often complex and generate large datasets, and the trials we reviewed were no exception [3]. Furthermore, the statistical report is a fundamental part of the research that has a crucial influence on what is written in the publication. Omission of a company statistician, usually also from the acknowledgment section, deprives readers of a key insight into the role of the company, although it is sometimes evident that reports of industry-sponsored trials contain sophisticated statistical analyses that are beyond the capabilities of the authors [8]. We cannot exclude the possibility that data analyses in some of the trials, and corresponding sections in protocols, were performed by company employees who were named authors but not statisticians, but it is unlikely since the pharmaceutical corporations usually have strong departments of statistics [8]. We believe it is wrong to deny a person who has contributed substantially (e.g., by performing the statistical analyses and by writing the statistical report) the opportunity to comment on the paper and finally approve of it, thereby fulfilling all three criteria for authors defined by the International Committee of Medical Journal Editors [4].

A potentially important reason for the missing company authors could be a change of job, as the median time span between protocol approval and publication was about five years. This should not be a valid reason for the former company to deny authorship to an individual, but companies have sometimes denied even their current employees deserved authorship [9], probably because of the perceived marketing advantage of papers that appear to have been written entirely by clinicians. However, if persons who qualify for authorship decline voluntarily, their contribution should be acknowledged according to guidelines for editors [4] and pharmaceutical companies [5,10]. Written permission to be acknowledged is usually required, however, which might explain some of the missing acknowledgments.

The guidelines on good publication practices for pharmaceutical companies [10] specify that whatever criteria for authorship are used, they should be applied in the same way to both external investigators and company employees. Furthermore, a company's involvement in data analysis and preparation of the manuscript should be made clear; publications should present the results accurately, objectively, and in a balanced fashion; and statisticians should participate in the preparation of publications. The International Committee of Medical Journal Editors has similar recommendations but does not give explicit advice on the role of statisticians [4]. Other guidelines have emphasized the need to acknowledge the role of medical writers (e.g., those for the World Association of Medical Editors [11] and European Medical Writers Association [12]).

For the most part, the current situation does not reflect these recommendations, and there are indications that they may be difficult to implement. First, legal proceedings and testimonies suggest that it is very common for professional medical writers to compose trial reports, reviews, and other papers for the pharmaceutical industry, but that their role is not revealed [13–18]. Companies and medical writing agencies may routinely disguise the fact that papers have been ghost-written [13], including erasing the file history of electronic documents before manuscript submission [19]. We found no references to medical writers in the protocols we reviewed, and they were acknowledged in only four publications (9%), which is consistent with a recent review of research articles [20]. Second, writing agencies have a vested interest in pleasing their clients by writing favourably about the drug in question [13–15,18,21]. Such commercial pressures may explain why conclusions in randomised trials recommended the experimental drug as the drug of choice much more often if the trial was funded by for-profit organisations, even after adjustment for the effect size (odds ratio 5.3) [22]. Third, honorary (guest) authorship for clinicians is very common [1,3,6,13,17,21]. Fourth, only six pharmaceutical companies have endorsed the guidelines for good publication practice for pharmaceutical companies that were published in 2003 [23]. In addition, 18 contract research and communications companies have agreed to recommend the guidelines to their clients and to follow them in their work, but such contractors might not be aware of omissions of qualifying authors and may not be able to convince their clients to comply.

We conclude that ghost authorship in industry-initiated randomised trials is very common, and we believe that this practice serves commercial purposes [13,17,21,22]. Its prevalence could be considerably reduced if existing guidelines were followed; in particular, journals should list the contributions of all authors [24]. In addition, journals could ask for the name and affiliation of the statistician who analysed the data, if this information is not clear. To improve transparency and accountability, there is also a need to specify in protocols who the statisticians and authors will be, and to make protocols and raw data from trials publicly available for independent analyses and interpretation [3,9,13,25]. This practice could increase the likelihood that publications accurately, fairly, and comprehensively reflect the collected data.
